# Establishing a Link Between Prescription Drug Abuse and Illicit Online Pharmacies: Analysis of Twitter Data

**DOI:** 10.2196/jmir.5144

**Published:** 2015-12-16

**Authors:** Takeo Katsuki, Tim Ken Mackey, Raphael Cuomo

**Affiliations:** ^1^ Kavli Institute for Brain and Mind University of California, San Diego La Jolla, CA United States; ^2^ Department of Anesthesiology UC San Diego - School of Medicine La Jolla, CA United States; ^3^ Division of Global Public Health UC San Diego - School of Medicine La Jolla, CA United States; ^4^ Global Health Policy Institute La Jolla, CA United States; ^5^ Joint Doctoral Program in Global Public Health UC San Diego - San Diego State University La Jolla, CA United States

**Keywords:** social media, surveillance, prescription drug abuse, twitter, eHealth, illicit Internet pharmacies, cyberpharmacies, infodemiology, infoveillance

## Abstract

**Background:**

Youth and adolescent non-medical use of prescription medications (NUPM) has become a national epidemic. However, little is known about the association between promotion of NUPM behavior and access via the popular social media microblogging site, Twitter, which is currently used by a third of all teens.

**Objective:**

In order to better assess NUPM behavior online, this study conducts surveillance and analysis of Twitter data to characterize the frequency of NUPM-related tweets and also identifies illegal access to drugs of abuse via online pharmacies.

**Methods:**

Tweets were collected over a 2-week period from April 1-14, 2015, by applying NUPM keyword filters for both generic/chemical and street names associated with drugs of abuse using the Twitter public streaming application programming interface. Tweets were then analyzed for relevance to NUPM and whether they promoted illegal online access to prescription drugs using a protocol of content coding and supervised machine learning.

**Results:**

A total of 2,417,662 tweets were collected and analyzed for this study. Tweets filtered for generic drugs names comprised 232,108 tweets, including 22,174 unique associated uniform resource locators (URLs), and 2,185,554 tweets (376,304 unique URLs) filtered for street names. Applying an iterative process of manual content coding and supervised machine learning, 81.72% of the generic and 12.28% of the street NUPM datasets were predicted as having content relevant to NUPM respectively. By examining hyperlinks associated with NUPM relevant content for the generic Twitter dataset, we discovered that 75.72% of the tweets with URLs included a hyperlink to an online marketing affiliate that directly linked to an illicit online pharmacy advertising the sale of Valium without a prescription.

**Conclusions:**

This study examined the association between Twitter content, NUPM behavior promotion, and online access to drugs using a broad set of prescription drug keywords. Initial results are concerning, as our study found over 45,000 tweets that directly promoted NUPM by providing a URL that actively marketed the illegal online sale of prescription drugs of abuse. Additional research is needed to further establish the link between Twitter content and NUPM, as well as to help inform future technology-based tools, online health promotion activities, and public policy to combat NUPM online.

## Introduction

Prescription drug abuse among youth and adolescents is a recognized national public health crisis [1]. Current data and behavioral trends on “non-medical use of prescription medication” (NUPM) are largely derived from nationally representative anonymous self-administered surveys that ask American high school students to self-report recent and past drug abuse behavior [2,3]. However, the rapid increase in Internet use, social media engagement, and near universal access to mobile devices among teens (aged 13-17) allows for augmentation of traditional NUPM survey data with other digital sources of information that are readily available for “big data” analysis and that can be used for health surveillance and prevention [4,5]. Specifically, Internet users are increasingly “self-reporting” their behavior on a variety of health subjects outside of structured surveys via multiple online social networking channels, including platforms such as Twitter, Facebook, Instagram, blogs, and other social sharing sites. In order to leverage this secondary source of information that can contribute to a better understanding of NUPM, this study identifies, characterizes, and describes prescription drug abuse trends and behavior via the popular microblogging platform, Twitter, which has already been associated with risky health behavior and is heavily populated by youth and adolescents [1,6,7].

Twitter currently commands some 316 million active monthly users and, though not the predominant social media site among teens, is used by an estimated one third of this age demographic and by 23% of all online adults, thereby serving as an important social and communication information-based research tool [4,8]. Additionally, compared to other social network platforms, Twitter provides one of the most versatile public application programming interfaces (APIs), allowing users to access large-scale real-time and historical communication data, though certain limitations in collecting such data exists (discussed below).

Hence, this study seeks to leverage the ability to access, construct, and analyze large conversational datasets from Twitter in order to assess how NUPM is being promoted in this environment of Internet users, similar to previous studies using Twitter to address other important public health issues, including drug safety [9-17]. The study also expands on prior studies assessing the association between NUPM and Twitter by examining whether the content of user-generated tweets directly enables NUPM access to prescription medications from illicit online pharmacies [7,10,18,19]. Illegal marketing and sales of prescription drugs by online pharmacies is an important public health and patient safety issue that the World Health Organization, US Food and Drug Administration, US Drug Enforcement Agency, and other stakeholders recognize as needing to be addressed [1,6,7,19].

## Methods

The methods for this study consist of two distinct phases: data collection (Phase 1) and data coding, analysis, and visualization (Phase 2). We describe each of these phases in detail below.

### Phase 1: Data Collection

**Figure 1:**
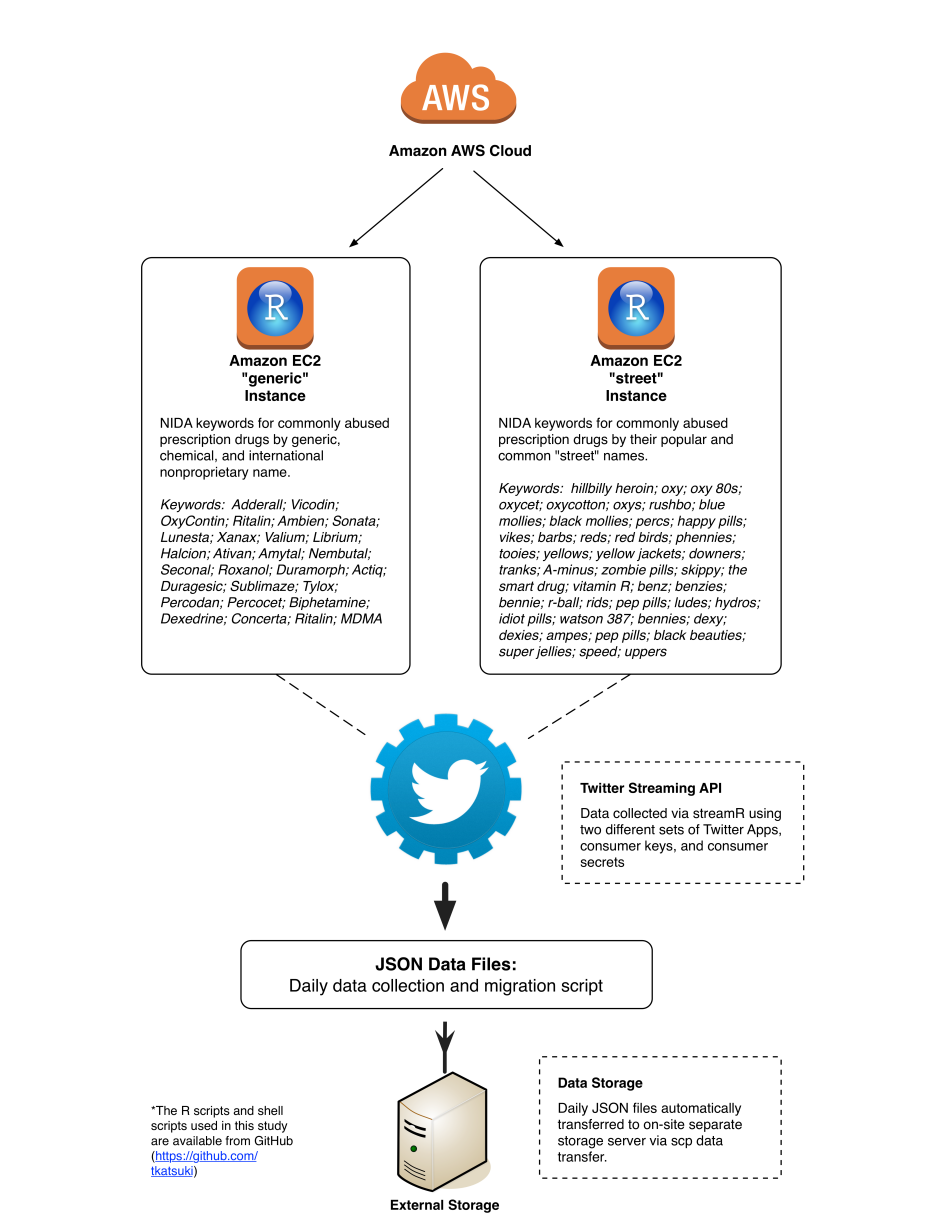
Data collection strategy.

Phase 1 of the study first identified prescription drugs commonly abused by youth and adolescents using information available from the National Institute on Drug Abuse and developed keywords as filters that were then applied to the collection of Twitter data (see Figure 1 for keywords used and visual depiction of the data collection strategy) [20]. We used the identified drug’s generic/chemical/international nonproprietary name (eg, oxycodone) and brand name (eg, OxyContin, Percocet) in one set of data collected (ie, Generic Names), and the common “street” or “slang” names (eg, oxy, oxycotton) of drugs in another set of data collection (ie, Street Names) in order to optimize conversational data capture associated with NUPM promotion and behavior [21,22]. Data were collected from the public Twitter Streaming API, and we applied the identified keywords/filters as endpoints in the data capture. This provided us with multiple raw JavaScript Object Notation (JSON) datasets of Twitter feeds and associated metadata for further analysis.

The study conducted an analysis of a 2-week subset of data collected and analyzed using this process from April 1-14, 2015 (ie, Study Data). The two separate datasets of tweets (one filtered for a drug’s generic name and a second for street names) were collected from the Twitter Streaming API using streamR package in R (CRAN), which was deployed on cloud-based computing services offered by Amazon Web Services (AWS) via Amazon EC2 t2.micro instances. In accessing the Twitter Streaming API, we used two different sets of Twitter apps, Consumer Keys (API Keys) and Consumer Secrets (API Secrets), in order to maximize data capture and lower the chance of hitting the Twitter Streaming API cap. The 2-week subset of data is part of a larger Twitter NUPM data mining project that has collected 3 months’ worth of data and that is undergoing separate analysis. AWS services were chosen due to their relative low cost (discussed below) and primarily for their stability in collecting, transferring, and storing data generated for this project. Specifically, the reliability of AWS (guaranteed availability of 99.95% for external connectivity) ensures contiguity of data when using multiple instances to collect data from the Twitter Streaming API.

R for streaming Twitter data was run on an RStudio Server preconfigured on an Amazon Machine Image (ami-45c72a01) originally developed and made freely accessible to the public (Louis Aslett’s RStudio Server Amazon Machine Image website). Streaming was scheduled to iteratively initiate and end every 24 hours, generating daily JSON files that included Twitter data filtered for prescription drug abuse keywords. In the event that streaming was interrupted for any reason, the script was written to automatically prompt the restart of the streaming collection process. The daily files were automatically transferred to a separate file storage server via SCP data transfer, and original files on the AWS server were deleted via SSH if the transfer was successful. Data analysis was performed on a local machine (Dell Precision T5810, 64GB memory, 4 CPU cores) or on an Amazon EC2 m4.4xlarge instance (64GB memory, 16 CPU cores). The R scripts and shell scripts used in this study are available from the first author's GitHub repository (TK).

### Phase 2: Data Coding, Analysis, and Visualization

Phase 2 of this study involved analyzing data for characteristics of interest by conducting data content coding using a supervised machine learning protocol. The process was first carried out by the second and third authors who acted as human coders and independently reviewed and coded a subset of 1000 randomly selected tweets from each instance. The second author, with expertise and training in substance abuse behavior, trained the third author for content coding. These randomly sampled tweets (including the textual content and select metadata) were reviewed and coded for the following characteristics: (1) relevance to NUPM behavior (ie, reviewing tweets and assessing if they actually discussed NUPM behavior and/or promotion) and (2) assessing NUPM characterization (positive or negative promotion/attitudes) (see Table 1 for details on content coding).

**Table 1:** Content analysis categories.

Relevant vs non-relevant	Favorable, non-favorable, neutral content analysis	Illicit online source information
Relevant: Contained content discussing NUPM behavior, attitudes, information about buying online, reporting health effects	Favorable promotion: Emphasizing benefits and/or minimizing risks regarding NUPM and generally promoting NUPM lifestyle/behavior	Online access: Providing a URL/hyperlink to “buy” or “online purchase” of prescription drugs
Non-relevant: Topics not associated with NUPM (eg, sports, consumer goods, news reports, music, lawful use of drugs in clinical settings) and tweets without sufficient content to code	Non-favorable promotion: Providing information on risk, side effects, or information on addiction treatment	Risk characteristics of online pharmacy linked to content: Online pharmacy identified as “unapproved” or “rogue” on Legitscript site^a^

^a^LegitScript: “rogue” is categorized as a website that appears to be intentionally or knowingly violating applicable laws or regulations; “unapproved” is categorized as verified as lacking compliance with LegitScript standards or other applicable laws and regulations.

This subset of human coded tweets was then used to train machine classifiers for “relevance” and “favorability” by a Support Vector Machine (SVM) algorithm in R that was then applied to the full dataset of collected tweets. Accuracy of the models was tested with 10-fold cross-validations with 3 repeats using the caret package [23]. In order to create a feature vector representation of each document (tweet), a corpus of the subsampled tweets was generated using the tm package in R [24]. This process involved data cleaning by transforming all texts to lower case, removing uniform resource locators (URLs), numbers, punctuations, and stop words (English language plus “re” and “rt”), as well as word grouping (n-grams) followed by generation of a Term-Document Matrix.

Qualitative analysis of tweets was then conducted by analyzing the source JSON Twitter data in streamR package that parses JSON files and transforms them in R data frames. Additional analysis was conducted by exporting JSON data to CSV (comma separated values) format and importing it into software NVivo 10 (QSR International) for further data storage, organization, and management. R was also used to visualize a word cloud associated with the highest frequency terms detected in Twitter content in order to better identify thematic categories in the data (“wordcloud” package).

We also manually coded the subset of tweets determined as relevant for NUPM for identification of any URLs/hyperlinks in tweets advertising online sale of prescription drugs. We used the NVivo NCapture feature to archive websites with hyperlinks in order to review content and determine if they were relevant to NUPM, acted as marketing affiliates, or directly sold prescription drugs online. A review of the risk status of illicit online pharmacy NUPM links was achieved by cross-referencing websites associated with hyperlinks using a database from the private Internet monitoring company, LegitScript, which provides information on websites likely in violation of applicable laws in one or more countries [25].

All tweets classified as NUPM-relevant that also had available latitude and longitude data were geocoded as individual points onto a contiguous map of the United States using ArcGIS version 10.1 (Esri). A publically available basemap was downloaded from the Esri website, and a scale of 1 centimeter:150 kilometers was applied. For the geographic coordinate system, we adopted the World Geodetic System 1984 standard. Zip code–level data on the number of individuals by age group was downloaded from the US Census Bureau, and the kernel density function was used to create a heatmap from this data, thereby displaying a gradient from blue to red for lower density of individuals between ages 15-19 to higher density of individuals in this age group, respectively. This was done to visualize the distribution of NUPM geocoded tweets to regions of the United States with a higher density of teens and young adults.

## Results

### Data Collection Results

We experienced no detectable interruptions in service during the 2-week study data period for our two separate Rstudio AWS instances. In total, our study data yielded 2,417,662 tweets, comprising 232,108 generic name tweets, 72.53% (n=168,355) of which were in English; and 2,185,554 street name tweets, 81.74% (n=1,786,626) of which were in English. Study data for the generic name instance yielded 1.44% (n=3351) that were geocoded for geographic location (a similar rate of location-enabled tweets compared to previous studies [26,27]), and 49.84% (n=115,685) that included URLs (comprising 22,174 unique URLs). Similarly, our street name NUPM instance yielded 1.39% (n=30,274) geocoded tweets and 43.51% (n=951,107) that included URLs (comprising 376,304 unique URLs). We describe preliminary data characteristics and their association with NUPM promotion, behavior, and access for both instances in the next section.

We note that during the study data period we received limit notices (limit count) for both instances collecting data from the public streaming API indicating that a filtered stream matched more tweets than the rate limit allowed to be delivered [28]. Twitter limit notices provide a total count of the number of undelivered tweets since the API connection was opened and can contextualize how representative filtered data collected from the Twitter public API is compared to the full Twitter firehose (which offers full and complete access to current and historic tweets). A total count of 247 tweets (corresponding to 0.1% of collected tweets) and 8327 (0.38%) in the generic and street name instances were reported, respectively.

### Word Frequency and Association

Prior to conducting our content coding for study data, we first conducted text mining and analysis in R using the tm package. After generating a Term-Document Matrix, we analyzed Twitter NUPM content for word frequency and association. The most frequently observed words contained in the generic name dataset (frequency count includes all words used in a tweet including non-keyword terms) were for several drugs subject to abuse by youth and adolescents including: Valium (6.23% of all words observed, n=50,769, rank #1), Adderall (5.33%, n=43,426, rank #3), and Xanax (4.48%, n=36,486, rank #4). The relative high frequency of specific NUPM keywords indicates that even without using a separate process for iteratively filtering and coding of Twitter content, the original filters applied in this instance were more likely to yield a reasonable percentage of NUPM relevant tweets given the specificity of keywords.

Conversely, in the street name instance, we immediately noticed that non-specific street names for drugs (primarily “speed,” which refers to amphetamine; “reds,” which refers to barbiturates; and “benz,” which refers to benzedrine) were used in several thousands of tweets unrelated to drug abuse. The presence of tweets that were “noisy” (ie, prescription drug abuse slang terms that introduce extreme noise as they are used in more common words or return results about unrelated topics) in this instance necessitated further filtering of the dataset, pointing to the need for better construction of keyword filters during the initial data collection phase [29]. Based on these preliminary results, we conducted a separate filtering of the street name dataset to exclude the above three keywords that introduced noise and used this new dataset of 79,949 tweets during the content analysis process. The words with the highest frequency in the street name instance were “skippy” (slang for the drug Adderall/Ritalin, 2.73% of all words observed, n=12,453, rank #1), “yellows” (slang for barbiturates, 2.08%, n=9511, rank #2), “uppers” (slang for amphetamines, 2.08%, n=9498, rank #3), “barbs” (slang for barbiturates, 1.35%, n=6168, rank #4), and “oxy” (slang for OxyContin/Oxycodone, 1.15%, n=5269, rank #5).

**Figure 2:**
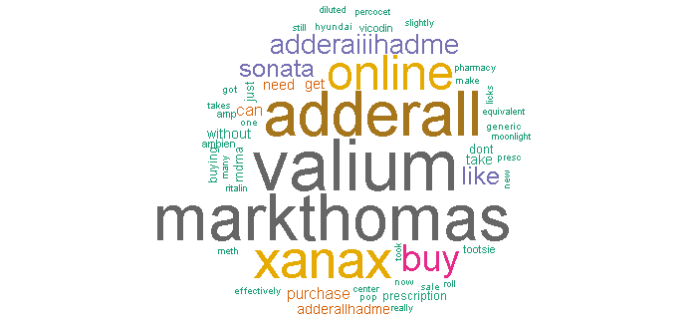
Word cloud for generic instance.

**Figure 3:**
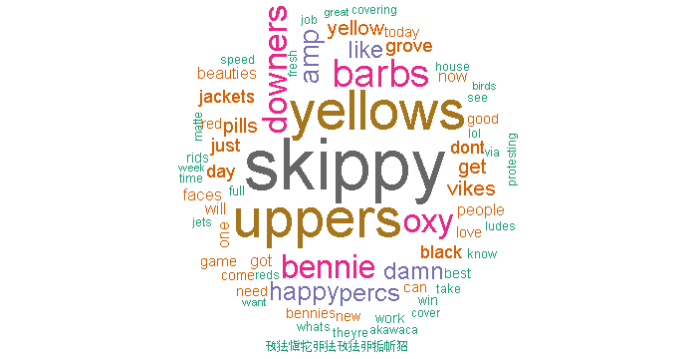
Word cloud for street instance.

Two separate word clouds were generated depicting the frequency of all words observed in the text of tweets analyzed for both instances (see Figures 2 and 3).

### Twitter NUPM Characteristics

In the next step, we applied our supervised machine learning content coding protocol in order to more specifically identify content relevant to NUPM. We used this process to filter out “false positive” tweets that were unrelated to NUPM but nevertheless were returned in filtered results as the keyword appeared somewhere in the text or metadata [22]. Coding of data between the 2 human coders achieved a high level of intercoder agreement for both subsamples of data from the two instances (all Cohen’s kappas for characteristics reviewed were greater than .83 and had a mean score of *k*=.91). Supervised machine learning was conducted by defining a feature space of documents (tweets) with a unigram term-document matrix. The accuracy score of the classifier models of tweet relevance to NUPM, when evaluated by repeating 10-fold cross validation three times, was 94.5% for the generic name dataset and 93.5% for the street name dataset, while accuracy scores of favorability for the generic name data were 95.1% and 94.1% for the street name data. We also compared the performance of models built from different word-grouping units (unigram, bigram, trigram, and combination of 1-3 grams); however, larger grouping units did not add significant performance improvement compared to unigram analysis. We therefore used the model created with unigram text data for the final analysis.

**Figure 4:**
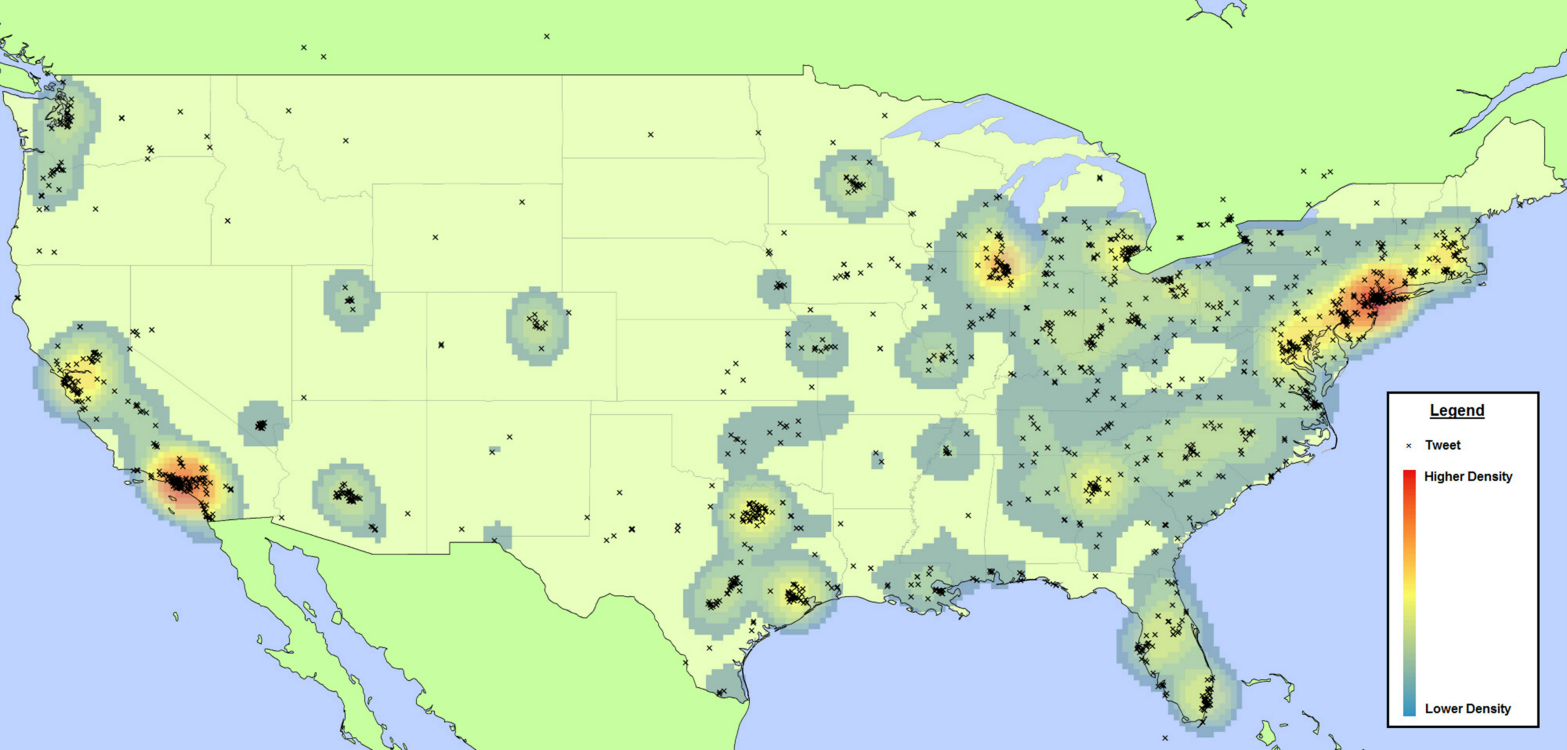
Geocoded NUPM relevant tweets by intensity and age demographics - United States.

Applying this classifier to the generic name dataset yielded a total of 135,776 tweets (81.72% of the English tweets with at least one keyword) that were predicted to be relevant to NUPM behavior or promotion. Conversely, false positives were detected in high frequency in the street name instance (eg, OXY, which is a stock listing symbol for the publicly traded company Occidental Petroleum Corporation and #uppers, which is a Twitter hashtag for users who engage in political discussion), with only 9817 (12.28%) tweets predicted as relevant to NUPM. Of the NUPM relevant tweets, an estimated 98.59% (n=133,863) and 78.76% (n=7732) favorably promoted NUPM behavior in the generic name and street name instances, respectively. Finally, after filtering both instances for NUPM-relevant tweets, 1.36% (n=1842) and 3.1% (n=308) were detected as geocoded, in the generic name and street name instances, respectively, with most of the tweets originating from users in California, Texas, and New York. In a map comparing individual geolocatable tweets with zip code–level kernel density of individuals aged 15-19, there was a positive relationship observed between NUPM tweets and areas with higher youth density (see Figure 4).

### Illegal Online Access to Prescription Drugs

**Figure 5:**
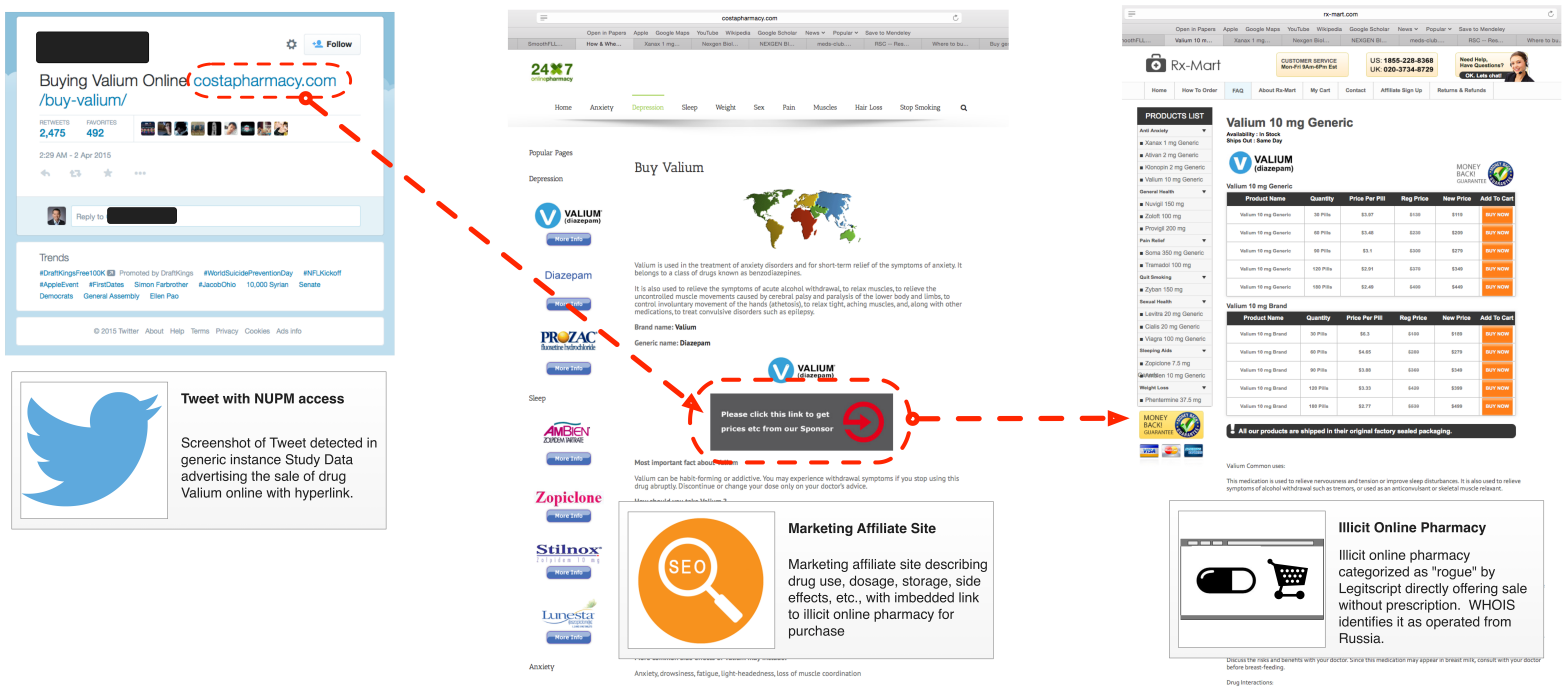
Twitter NUPM content illicit online pharmacy relationship.

In the NUPM relevant tweets for the generic name dataset, there were 59,845 hyperlinks with 5039 unique URLs. The vast majority of these hyperlinks (88.54%, n=52,988) were tweeted at least 9 times. We then manually reviewed the websites connected to hyperlinks for content tweeted at least 9 times in order to determine if websites actually marketed or sold prescription drugs. The most frequently observed URL was for a purported online pharmacy using the name “CostaPharmacy.” It was mentioned in 75.72% (n=45,317) of all tweets with hyperlinks and on further inspection was identified as an online marketing affiliate site that promoted the online purchase of Valium (diazepam), a benzodiazepine type of medication used as a tranquilizer that is commonly abused. A total of 8171 different Twitter users tweeted or retweeted to this affiliate site that contained, within its website content, a direct link to an illegal online pharmacy. Importantly, the linked online pharmacy was categorized as “rogue” by LegitScript indicating that the website appears to be intentionally or knowingly violating applicable laws or regulations. On review of WHOIS records, it appears to be located in Russia (see Figure 5 for visualization of tweet, hyperlinks, and connection between websites). By counting the number of users following accounts that mentioned the URL, we estimate that content containing the hyperlink connected with this illegal online pharmacy was broadcast to over 250,000 total Twitter users within the 2-week study data period. We also observed a much smaller number of additional tweets with hyperlinks to NUPM access points that either advertised the sale of a prescription drug and then linked to another site or actually claimed to sell the drug directly to customers via their online storefront. All associated links to online pharmacy sites reviewed were categorized as “rogue.” Additionally, other links we observed promoted NUPM but were not associated with an online pharmacy. This included Twitter content with links to hip hop songs promoting the NUPM lifestyle, NUPM promotional items sold on eBay, and a tweet linking to a website selling nutritional supplements advertised as substitutes for NUPM drugs (see Table 2 for examples).

**Table 2:** Examples of different categories of NUPM tweets with website links.

Category	Tweet content	Description
Lifestyle: individual user	RT @[ANONYMIZED]: "I wanna try xanax" Me https://t.co/XpTErzo6W6	Retweet of individual user promoting drug abuse initiation with link to video where user shows pills in hand
Lifestyle: individual user (polydrug mention)	RT @[ANONYMIZED]: Prescription drugs, show me lovePercocets, Adderall, Xanny bars, get codeine involved	Tweet describing promotion of polydrug abuse with several different therapeutic classes
Lifestyle: music	RT @[ANONYMIZED]: Go do some xanax, crank this shithttps://t.co/9N72OXO0rs	Retweet with link to streaming hip hop song promoting prescription drug abuse behavior
Commercial: individual seller	Check out Xanax Pill Necklace http://t.co/IfeGqe01t4 via @eBay	Link to eBay seller account for a Xanax pill bar necklace that promotes NUPM lifestyle
Commercial: company	Over the counter? #Adderall?#Xanaxsubstitute #anxietynaturalremedy? #herebalremedy http://ow.ly/MsyqQ	Tweet linking to website selling nutritional supplements that are advertised as substitutes to Adderall and Xanax
Online pharmacy–related link	RT @[ANONYMIZED]: How to Buy Valium Online http://t.co/qkDY8ZJ08W	Retweet with link to marketing affiliate that included a link to an illicit online pharmacy

We note that in the street name dataset, no NUPM online access links were detected. This was determined by manually reviewing all 653 unique URLs included in the NUPM relevant tweets for the street name dataset, revealing that none of the links were associated with an online pharmacy.

### Overall Cost and Accessibility

Important in assessing the feasibility of this study is describing the overall cost of project implementation. Primarily, we attempted to conduct the data collection phase of this study with computational and software resources already available in the public domain. This included the use of the R programming language as a data collection and analysis platform, which utilized several software packages that were open access and free to use. Since the Twitter Streaming API collects data in real-time, contiguity of data primarily depends on the reliability of the network connectivity. We therefore used AWS services to ensure fidelity of our data collection (ie, service level guarantees of 99.95% uptime). Overall, AWS services to support this study were delivered at low cost, at a total expense of US $17 per instance with 40GB of storage space (excluding the AWS free Tier of 750 machine hours per month of t2.micro instances) for a total data collection period of approximately 3 months (analysis for the entire period of data collection is ongoing). Because fees for AWS services are variable based upon size of storage, CPU utilization, and memory size, by regularly transferring data to an external storage space one can also minimize the cost required for data storage directly on AWS.

## Discussion

### Principal Results

Based on our analysis of the study data collected, it appears that NUPM promotion via Twitter is occurring frequently for specific keywords associated with a drug’s chemical or generic name and yields large-scale datasets that require the appropriate combination of analytical and computing tools to appropriately assess potential behavioral risk factors and online access. In comparison, the more indistinct use of NUPM street or popular names that includes descriptive or non-specific terms introduced more noise and far fewer relevant results compared to the generic name dataset. From a data collection standpoint, our 2-week period of study data yielded over 2 million tweets from both instances, with a total of 6.02% (n=145,593) determined relevant to NUPM behavior or promotion based on our use of supervised machine learning for content analysis.

Our most concerning finding was that in the generic name instance, a significant percentage (33.37%, n=45,317) of the machine classified NUPM relevant content originated from a highly propagated live hyperlink to a marketing affiliate that provided direct access to prescription drugs of abuse through an illegal online pharmacy. The marketing affiliate site advertises the online sale of several drugs of abuse, including Ativan, Ambien, Lunesta, Valium, and Xanax, among other classes of prescription drugs. The tweets for this hyperlink varied slightly in content, but all blatantly advertised the sale of Valium (eg, “RT @[username anonymized]: Valium Online Without Prescription [URL]” or “RT @[username anonymized]: Where to Buy Valium Online [URL]”) and were retweeted by a large network of Twitter accounts potentially exposing hundreds of thousands of Twitter users to NUPM promotion and access. As a Drug Enforcement Agency Schedule IV controlled substance, the online sale of Valium and other drugs that carry the potential for abuse and dependence, is in direct violation of the Ryan Haight Online Pharmacy Consumer Protection Act, a federal law named after a San Diego Teen who lost his life after overdosing from illegally purchasing prescription pain killers online [1,6].

Finally, the relatively low count in undelivered tweets compared to the total tweets collected in each instance may indicate that our data collection methodology has a higher rate of completeness (99.9% for the generic name instance and 99.7% for the street name instance) and is a more representative sample compared to what is generally represented in the literature regarding the Twitter Public API [22]. It may also indicate that by creating separate virtual instances for data collection operating on different keyword filters and consumer keys/access tokens, our data collection process can avoid Streaming API rate limits (usually estimated at 1% sample of all tweets). Overall, the study supports findings from prior studies that have used the Twitter Streaming API to collect large amounts of Twitter content instead of licensing content from a third-party data reseller that may be cost-prohibitive to certain researchers (ie, the starting price for a GNIP Twitter dataset request is US $1250) [13,26,30,31].

### Limitations

There are certain limitations to our study that impact the generalizability of results to NUPM behavior and promotion via Twitter. When hand coding tweets, human coders observed that some tweets were extremely short (ie, 1-2 words) and did not contain content that indicated that it promoted NUPM behavior or access, even though the keyword was contained in the tweet. These tweets were coded as non-relevant by human coders as they would likely be interpreted by Twitter users as non-relevant and were a very small percentage (˂5%) of overall human coded tweets. Additionally, the text of some tweets contained hyperlinks to images and other media associated with the tweet that helped contextualize the content/message or confirm promotion of NUPM behavior. Although human coders reviewed these images that were linked to these tweets, which aided in their interpretation of content classification, our machine learning algorithm was not able to analyze this media in subsequent machine classification prediction. We also estimated the number of Twitter users who potentially received the rogue online pharmacy Valium hyperlink by examining the followers_count field of the user statuses. Although this approach is limited in that the analysis can be performed only retrospectively and thus the followers_count may differ from the time the tweets were actually generated, the result is indicative of how a Twitter user network can play a role in promoting illicit online pharmacies to a broad base of users.

Finally, given the large scale of data collected per instance relative to the short time period examined, future content coding of NUPM Twitter data will likely need to be assisted with additional high-performance computational tools/services in order to make such a project scalable over a longer period of data collection. Further, more iterative rounds of human coding using Twitter data collected over a longer period of time with more diversity in Twitter users and content could help improve the machine learning process. Possible solutions to augment trained human coding include the use of crowdsourcing large networks of human coders (eg, Amazon Mechanical Turk workers) or the use of new content coding services in the cloud, such as those offered by the company DiscoverText, that offer cloud-based text analytics solutions targeted for analyzing social media data [32,33]. These tools and platforms have already been utilized in previous substance abuse studies and could be applied to future work analyzing a larger dataset of NUPM tweets [11,32-34]. We also did not filter for language as we are considering the possibility of using language information in future studies. Here, however, we content coded only English tweets and not non-English tweets (19.14%, n=462,681 of the dataset.) We also encountered a handful of “dead” links in the hyperlinks manually coded for association with an illicit online pharmacy, though we note in both cases this was an extremely small percentage of the total tweets collected over the study period.

### Comparison With Prior Work

The few studies that have specifically examined the association between Twitter and NUPM have focused on testing the ability to illegally advertise illicit online pharmacy content via a fictitious Twitter account, qualitatively assessing tweets about prescription opioids, online social engagement between networks of prescription drug abusers that use Twitter, and Twitter use to promote drug abuse of Adderall among college students [7,10,19,35]. This study expands on previous studies to further explore how the Twitter environment can promote NUPM behavior and access by examining a broad set of prescription drug keywords associated with abuse by youth and adolescents. The study builds on previous research that has used different sets of prescription drug and common/slang keywords to filter and analyze Twitter data, as well as prior studies that have analyzed the content of hyperlinks detected in large-scale datasets of filtered tweets for other public-health related topics [16,36-38].

### Conclusions

As youth and adolescents increasingly engage in online communities, social relationships, and conversations about NUPM via popular social media platforms such as Twitter, additional research is critical in order to leverage strategies of “infoveillance” to collect data needed to tailor future public health interventions attempting to combat prescription drug abuse among this vulnerable population [5,39]. Importantly, analysis of real-time data via Twitter, can help inform and contextualize traditional public health surveillance approaches collected through national surveys and also help proactively identify changing and emerging trends in prescription drug abuse behavior that are unique to the online environment. The study also identifies Twitter as a potential source for information illegally promoting the sale of controlled prescription drugs directly to consumers, which is a concerning observation given the inherent risk of abuse, dependency, and questionable authenticity of medicines provided by online pharmacies who are in violation of applicable law, including the US Ryan Haight Act. These results support renewed focus to better understand these understudied channels of NUPM promotion and needed commitment to develop technology-based tools, online health promotion activities, and public policy protecting youth and adolescents from prescription drug abuse online.

Acknowledgments

TK and TM received funding for this research from the Alliance for Safe Online Pharmacies (ASOP), a 501(c)(4) social welfare organization engaged in the issue of illicit online pharmacies, and greatly acknowledge this support. The funder had no role or input in the study.

## Abbreviations

API: application programming interface
ASOP: Alliance for Safe Online Pharmacies
AWS: Amazon Web Services
JSON: JavaScript Object Notation
NUPM: non-medical use of prescription medication
SVM: Support Vector Machine (algorithm)
URL: uniform resource locator

## References

[1] Mackey TK, Liang BA, Strathdee SA (2013). Digital social media, youth, and nonmedical use of prescription drugs: the need for reform. J Med Internet Res.

[2] McCabe SE, West BT, Teter CJ, Boyd CJ (2012). Co-ingestion of prescription opioids and other drugs among high school seniors: results from a national study. Drug Alcohol Depend.

[3] Inciardi JA, Surratt HL, Cicero TJ, Rosenblum A, Ahwah C, Bailey JE, Dart RC, Burke JJ (2010). Prescription drugs purchased through the internet: who are the end users?. Drug Alcohol Depend.

[4] Lenhard A (2015). Pew Internet Research.

[5] Stoové MA, Pedrana AE (2014). Making the most of a brave new world: opportunities and considerations for using Twitter as a public health monitoring tool. Prev Med.

[6] Liang BA, Mackey T (2009). Searching for safety: addressing search engine, website, and provider accountability for illicit online drug sales. Am J Law Med.

[7] Hanson CL, Cannon B, Burton S, Giraud-Carrier C (2013). An exploration of social circles and prescription drug abuse through Twitter. J Med Internet Res.

[8] Duggan M, Ellison N, Lampe C, Lenhart A, Madden M (2015). Pew Internet Research.

[9] Ghosh DD, Guha R (2013). What are we 'tweeting' about obesity? Mapping tweets with Topic Modeling and Geographic Information System. Cartogr Geogr Inf Sci.

[10] Hanson CL, Burton SH, Giraud-Carrier C, West JH, Barnes MD, Hansen B (2013). Tweaking and tweeting: exploring Twitter for nonmedical use of a psychostimulant drug (Adderall) among college students. J Med Internet Res.

[11] Huang J, Kornfield R, Szczypka G, Emery SL (2014). A cross-sectional examination of marketing of electronic cigarettes on Twitter. Tob Control.

[12] Bosley JC, Zhao NW, Hill S, Shofer FS, Asch DA, Becker LB, Merchant RM (2013). Decoding twitter: Surveillance and trends for cardiac arrest and resuscitation communication. Resuscitation.

[13] Myslín M, Zhu S, Chapman W, Conway M (2013). Using twitter to examine smoking behavior and perceptions of emerging tobacco products. J Med Internet Res.

[14] O'Connor K, Pimpalkhute P, Nikfarjam A, Ginn R, Smith KL, Gonzalez G (2014). Pharmacovigilance on twitter? Mining tweets for adverse drug reactions. AMIA Annu Symp Proc.

[15] Freifeld C, Brownstein J, Menone C, Bao W, Filice R, Kass-Hout T, Dasgupta N (2014). Digital drug safety surveillance: monitoring pharmaceutical products in twitter. Drug Saf.

[16] Carbonell P, Mayer MA, Bravo A (2015). Exploring brand-name drug mentions on Twitter for pharmacovigilance. Stud Health Technol Inform.

[17] Broniatowski DA, Paul MJ, Dredze M (2013). National and local influenza surveillance through Twitter: an analysis of the 2012-2013 influenza epidemic. PLoS One.

[18] Dasgupta N, Freifeld C, Brownstein JS, Menone CM, Surratt HL, Poppish L, Green JL, Lavonas EJ, Dart RC (2013). Crowdsourcing black market prices for prescription opioids. J Med Internet Res.

[19] Mackey TK, Liang BA (2013). Global reach of direct-to-consumer advertising using social media for illicit online drug sales. J Med Internet Res.

[20] NIDA (2015). drugabuse.

[21] Yoon S, Elhadad N, Bakken S (2013). A practical approach for content mining of Tweets. Am J Prev Med.

[22] Kim AE, Hansen HM, Murphy J, Richards AK, Duke J, Allen JA (2013). Methodological considerations in analyzing Twitter data. J Natl Cancer Inst Monogr.

[23] Kuhn M (2008). Building predictive models in R using the caret package. J Stat Soft.

[24] Feinerer I, Hornik K, Meyer D (2008). Text Mining Infrastructure in R. J Stat Soft.

[25] LegitScript.com.

[26] Nagar R, Yuan Q, Freifeld CC, Santillana M, Nojima A, Chunara R, Brownstein JS (2014). A case study of the New York City 2012-2013 influenza season with daily geocoded Twitter data from temporal and spatiotemporal perspectives. J Med Internet Res.

[27] Burton SH, Tanner KW, Giraud-Carrier CG, West JH, Barnes MD (2012). "Right time, right place" health communication on Twitter: value and accuracy of location information. J Med Internet Res.

[28] (2015). Streaming message types.

[29] Adrover C, Bodnar T, Huang Z, Telenti A, Salathé M (2015). Identifying Adverse Effects of HIV Drug Treatment and Associated Sentiments Using Twitter. JMIR Public Health Surveill.

[30] Zhang N, Campo Shelly, Janz Kathleen F, Eckler Petya, Yang Jingzhen, Snetselaar Linda G, Signorini Alessio (2013). Electronic word of mouth on twitter about physical activity in the United States: exploratory infodemiology study. J Med Internet Res.

[31] McIver DJ, Hawkins JB, Chunara R, Chatterjee AK, Bhandari A, Fitzgerald TP, Jain SH, Brownstein JS (2015). Characterizing Sleep Issues Using Twitter. J Med Internet Res.

[32] Paul MJ, Dredze M (2014). Discovering health topics in social media using topic models. PLoS One.

[33] Choundhury MD, Gamon M, Counts S, Horvitz E (2013). Predicting Depression via Social Media. Seventh International AAAI Conference on Weblogs and Social Media.

[34] Choundhury MD, Counts S, Horvitz E (2013). Social Media as a Measurement Tool of Depression in Populations.

[35] Shutler L, Nelson LS, Portelli I, Blachford C, Perrone J (2015). Drug Use in the Twittersphere: A Qualitative Contextual Analysis of Tweets About Prescription Drugs. J Addict Dis.

[36] Harris J, Moreland-Russell S, Choucair B, Mansour R, Staub M, Simmons K (2014). Tweeting for and against public health policy: response to the Chicago Department of Public Health's electronic cigarette Twitter campaign. J Med Internet Res.

[37] Prochaska JJ, Pechmann C, Kim R, Leonhardt JM (2012). Twitter=quitter? An analysis of Twitter quit smoking social networks. Tob Control.

[38] Lee JL, DeCamp M, Dredze M, Chisolm MS, Berger ZD (2014). What are health-related users tweeting? A qualitative content analysis of health-related users and their messages on twitter. J Med Internet Res.

[39] Nelson L, Meisel Z, Perrone J (2015). Opportunities for Exploring and Reducing Prescription Drug Abuse Through Social Media. J Addict Dis.

